# Implication of Vegetable Oil-Derived Hydroxynonenal in the Lysosomal Cell Death for Lifestyle-Related Diseases

**DOI:** 10.3390/nu15030609

**Published:** 2023-01-24

**Authors:** Tetsumori Yamashima

**Affiliations:** 1 Department of Psychiatry and Behavioral Science, Kanazawa University Graduate School of Medical Sciences, Kanazawa 920-8640, Japan; yamashima215@gmail.com; 2 Department of Cell Metabolism and Nutrition, Kanazawa University Graduate School of Medical Sciences, Kanazawa 920-8640, Japan

**Keywords:** calpain-cathepsin hypothesis, carbonylation, electron microscopy, Hsp70.1, hydroxynonenal, lysosomal rupture, lysosomal membrane permeabilization, lifestyle-related diseases, ω-6 PUFA

## Abstract

Lysosomes are membrane-bound vesicular structures that mediate degradation and recycling of damaged macromolecules and organelles within the cell. For ensuring the place of degradation within the acidic organelle, the integrity of the lysosomal-limiting membrane is critical in order to not injure the cell. As lysosomes fade away in response to acute intense insults or long-term mild insults, dissolving lysosomes are hardly detected during the phase of cell degeneration. If observed at the right time, however, lysosomal membrane rupture/permeabilization can be detected using an electron microscope. In both the experimental and clinical materials, here the author reviewed electron microphotographs showing disintegrity of the lysosomal-limiting membrane. Regardless of insults, cell types, organs, diseases, or species, leakage of lysosomal content occurred either by the apparent disruption of the lysosomal membrane (rupture) and/or through the ultrastructurally blurred membrane (permeabilization). Since lysosomal rupture occurs in the early phase of necrotic cell death, it is difficult to find vivid lysosomes after the cell death or disease are completed. A lipid peroxidation product, 4-hydroxy-2-nonenal (hydroxynonenal), is incorporated into the serum by the intake of ω-6 polyunsaturated fatty acid-rich vegetable oils (exogenous), and/or is generated by the peroxidation of membrane lipids due to the oxidative stress (intrinsic). Exogenous and intrinsic hydroxynonenal may synergically oxidize the representative cell stress protein Hsp70.1, which has dual functions as a ‘chaperone protein’ and ‘lysosomal stabilizer’. Hydroxynonenal-mediated carbonylation of Hsp70.1 facilitates calpain-mediated cleavage to induce lysosomal membrane rupture and the resultant cell death. Currently, vegetable oils such as soybean and canola oils are the most widely consumed cooking oils at home and in restaurants worldwide. Accordingly, high linoleic acid content may be a major health concern, because cells can become damaged by its major end product, hydroxynonenal. By focusing on dynamic changes of the lysosomal membrane integrity at the ultrastructural level, implications of its rupture/permeabilization on cell death/degeneration were discussed as an etiology of lifestyle-related diseases.

## 1. Introduction

Lysosomes digest, recycle, and dispose of aged/damaged organelles, macromolecules, or proteins within the cell in both physiological and pathological conditions. A limiting membrane seals off their hydrolytic enzymes and prevents leakage into the cytoplasm in order to not damage the cell. In the early 1950s, Christian de Duve and his colleagues demonstrated that the lysosomal acid phosphatase is not detectable within the fresh cell homogenates, but can be detected in large quantities after cell fractionation through the differential centrifugation method. Furthermore, with the aid of an electron microscope, they observed numerous membrane-bound, tiny organelles in the cell fraction. These findings led them to speculate that acid phosphatase must be sealed in a sac, but it may break and release its content in response to severe insults. This phantom enzyme provided de Duve a clue to the serendipitous discovery of the ‘lysosome’ [1,2,3]. 

In the late 1960s, considerable interest was focused on the implication of lysosomal involvement in cell degeneration and necrosis. In 1966, de Duve and Wattiaux reported the concept that lethal cell injury occurs in pathological states by the release of hydrolytic enzymes from damaged lysosomes. They suggested that it might be an early and triggering event for cell injury [4,5]. In 1972, Brunk and Ericsson found that significant amounts of lysosomal acid phosphatases leak through the ultrastructurally intact lysosomal membrane in cultured glioma cells [6]. Subsequently, Brunk and his colleagues established the concept of lysosomal membrane permeabilization (LMP) in a series of works using cultured cells which were exposed to artificial oxidative stress [7,8,9,10,11]. They thought that acid phosphatases or other lysosomal enzymes may leak through the ultrastructurally intact lysosomal membrane, but not from the rupture sites at the membrane. The concept of LMP was widely accepted thereafter [12]. Therefore, for a long time, the lysosome has been imprecisely considered a sturdy organelle that does not disintegrate until the cell is already devitalized [13]). However, in 1996, Yamashima et al. found through electron microscopy that the rupture of the lysosomal-limiting membrane associated with the extra-lysosomal release of cathepsin enzymes occurs in the programmed necrosis of hippocampal *cornu Ammonis* 1 (CA1) neurons of macaque monkeys a few days after transient global brain ischemia [14]. This was the first report of lysosomal rupture, because the concept of apoptosis had been prevailing in the 1990s as a mechanism of programmed cell death.

Nowadays, it is believed that a low level of cell stress causes LMP and apoptosis, whereas a high level of cell stress causes lysosomal membrane rupture and necrosis [15,16,17]. LMP was suggested to induce apoptosis by mitochondrial transmembrane potential loss or caspase activation. In contrast, lysosomal membrane rupture induces necrosis due to the extensive leakage of cathepsin enzymes [10,11,13,14,18,19,20,21,22,23]. Traditionally, necrotic cell death was considered a passive death mode, which is triggered by catastrophic events such as heat shock, ischemia, irradiation, or irreparable stress to the cell [24]. More recently, many of these ‘unregulated’ cell death events were found to be essentially ‘regulated’, and the ‘*calpain-cathepsin hypothesis*’ was formulated to explain the mechanism of programmed neuronal necrosis in 1998 [25]. 

Lipid peroxidation product 4-hydroxy-2-nonenal (hydroxynonenal) is generated during deep-frying in cooking oils and/or within biomembranes by the long-standing oxidative stress. It may be either protective or damaging to the cells, depending on its concentration [26]. For example, at low concentrations, hydroxynonenal is involved in the control of signal transduction, gene expression, cell proliferation, differentiation, and cell cycle regulation. At high concentrations, however, hydroxynonenal forms adducts with proteins, nucleic acids and membrane lipids, which leads to long-term cell disorder and tissue damage [27,28]. Lifestyle-related diseases are defined as health disorders which are linked to the way people live their life. Representative diseases that are affected by the lifestyle are chronic diseases such as Alzheimer’s disease, type 2 diabetes, nonalcoholic steatohepatitis (NASH), ischemic heart diseases, etc. Since the etiology of these lifestyle-related diseases is complex and varies among individuals, identification of the most essential causative substances is very difficult, especially if their relative effect, i.e., acute toxicity, is weak and many years are needed for the disease progression due to chronic toxicity. Deep-frying using vegetable oils is a popular culinary method used worldwide, but the high oil content is a major health concern. For example, in the United States, nearly half of potatoes produced are deep-fried and processed into chips and French fries [29]. Hydroxynonenal is recently thought to be a key factor for damaging cells of the brain, pancreas, and liver [18,23,30,31]). However, the causative substance of lifestyle-related diseases, if present, still remains unknown. Recently, Yamashima et al. reported the occurrence of lysosomal membrane rupture/permeabilization in the neurons, Langerhans islet cells, and hepatocytes of monkeys after consecutive injections of synthetic ‘hydroxynonenal’. They speculated that the ω-6 polyunsaturated fatty acid (PUFA)-peroxidation product, which is contained in deep-fried foods cooked by linoleic acid-rich cooking oils, is the most suspicious causative substance of lifestyle-related diseases [18,23,30,31].

The contribution of lysosomes to diseases may be either active as a cause of cell death or passive as a consequence of cell death [32]. Here, the author provided a review of lysosomal membrane rupture/permeabilization as a cause of cell death/degeneration in the brain, heart, liver, and pancreas. Electron microscopic data of representative experimental paradigms and human diseases were introduced, such as (1) cultured cells exposed to oxidative stress, (2) *Caenorhabditis elegans* (*C. elegans*) *scav-*3 mutation models, (3) NASH model mice, (4) monkeys after consecutive hydroxynonenal injections, (5) monkeys after transient brain ischemia, and (6) human patients with Alzheimer’s disease or NASH. Interestingly, regardless of the insults, species, organs, or diseases, similar lysosomal membrane rupture/permeabilization was consistently observed as a cause of cell degeneration/death. This review aimed to raise concerns that the vegetable oil-derived hydroxynonenal may facilitate lysosomal cell death, and contribute to the occurrence of lifestyle-related diseases.

## 2. Cultured Cells Exposed to Oxidative Stress

**Figure 1:**
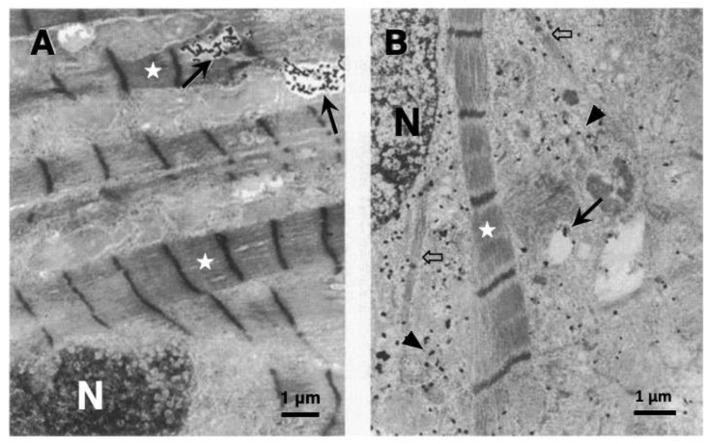
Immunoelectron microphotographs of rat heart myocytes showing immunoreactivity of cathepsin D. The control cell (**A**) shows that cathepsin-positive immunogold particles are localized within lysosomes ((**A**) arrows). In contrast, the cell exposed to 5 μmol/L naphthazarin for 30 min shows leakage of gold particles from the lysosomes into the cytoplasm ((**B**) arrow heads). The oxidative stress caused a remarkable loss of myofilaments ((**A**,**B**) stars) presumably by the extra-lysosomal release of cathepsin enzymes. Dissolving myofilaments ((**B**) open arrows) are surrounded by aggregated immunogold particles labelling cathepsin D. Lysosomes after the leakage of contents ((**B**) arrow) show a marked loss of immunogold particles, compared to those of the control ((**A**) arrows). This is evidence of lysosomal membrane permeabilization/rupture. N; nucleus. (Adapted from [35]).

The concept of LMP was first reported half a century ago in cultured cells treated with lysosomotropic detergents [6]. However, the interest in implicating lysosomes in necrotic cell death faded during the following two decades, and, concomitantly, lysosomal involvement in necrosis has been overlooked. This is presumably because the ability of caspase inhibitor Z-Val-Ala-Asp-fmk (zVAD-fmk) to inhibit cell death was considered as proof of not “necrosis” but “apoptosis”. Furthermore, many researchers had failed to detect LMP by conventional electron microscopic observation, because the lysosomal membrane looked grossly intact [33]. So, the presence of LMP was suggested initially not by conventional electron microscopy but by immunoelectron microscopic analysis. By analyzing the cytotoxicity of oxidized low-density lipoprotein to cultured macrophages, Li et al. (1998) observed extra-lysosomal release of cathepsin D using immunoelectron microscopic analysis [34]. Similarly, by exposing the cultured myocytes of a neonatal rat heart to oxidative stress using redox cycling quinone naphthazarin, Roberg and Öllinger (1998) observed both leakage of intra-lysosomal cathepsin D into the cytoplasm and dissolution of myofilaments through immunoelectron microscopic analysis (Figure 1) [35].

**Figure 2:**
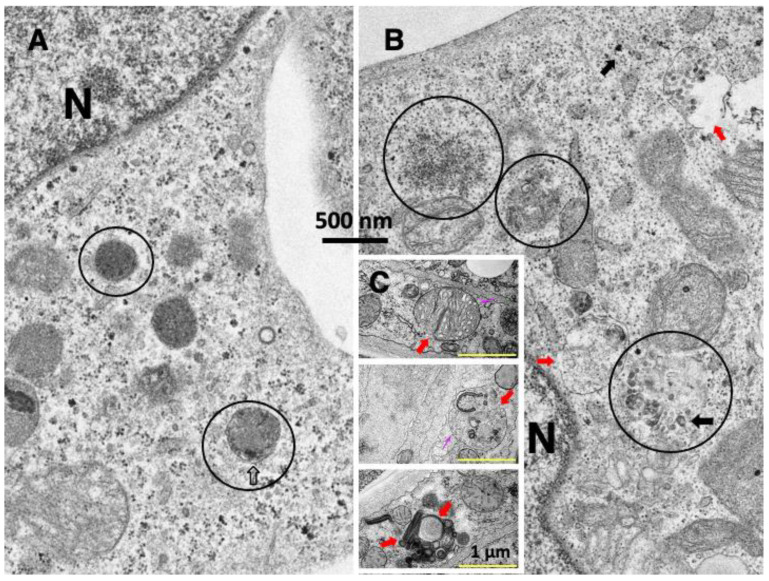
Electron microphotographs of hepatocellular carcinoma cell lines after hydroxynonenal treatment. Although lysosomes of the control cells (**A**) are bound by the distinct limiting membrane ((**A**) circles, open arrow), those of the carcinoma cells after hydroxynonenal treatment show partial ((**B**) red arrows) or total ((**B**) circles) disruptions of the lysosomal-limiting membrane with the resultant release of the content ((**B**) black arrows). Loss of *scav-*3*,* the *Caenorhabditis elegans* homologue of human LIMP-2 as a key regulator of lysosome integrity, caused similar rupture of the lysosomal-limiting membrane ((**C**) red arrows). Pink arrows show the intact portion of the limiting membrane, whereas red arrows show ruptured sites of membrane at lysosomes N; nucleus. ((**C**) adapted from [38]).

By exposing the cultured hepatocarcinoma cells to the synthetic hydroxynonenal, Seike et al. recently observed lysosomal membrane rupture using conventional electron microscopic analysis (Figure 2B, red arrows) [18]. The fluorescence time-lapse imaging of the HepG2 hepatocarcinoma cell lines clearly showed a rapid loss of lysosomes (being stained orange by LysoTracker) at the early phase of cell necrosis (Supplementary Movie, accessed 1 November 2022). This is the first animation which demonstrated an implication of lysosomal rupture in the occurrence of necrotic cell death [18]. The addition of hydroxynonenal in the cultured medium induced ‘bursting’ necrosis which was closely associated with the gradual reduction and loss of lysosomes in the early phase. Hydroxynonenal induced neither apoptotic bodies nor cell blebbings, but shrinkage of both the nucleus and the cytoplasm was observed. In contrast, the addition of an anti-cancer agent, epirubicin hydrochloride, in the medium induced apoptotic cell death with the formation of cell blebbings and apoptotic bodies, but lysosomes remained intact until the final phase, just prior to the cell death. Obviously, hydroxynonenal induced lysosome-mediated necrosis, whereas epirubicin hydrochloride induced lysosome-unrelated apoptosis.

## 3. *C. elegans scav-*3 Mutation Models

Human lysosomal integral membrane protein type 2 (LIMP-2, also known as SCARB2) is a lysosomal membrane protein that plays a crucial role in transporting β-glucocerebrosidase or cholesterol to lysosomes [36,37]. SCAV-3 is the *C. elegans* homologue of human LIMP-2 which serves as one of the key regulators of lysosomal membrane integrity. Li et al. reported that the loss of the *scav-*3 gene in *C. elegans* causes rupture of the lysosomal-limiting membrane [38]. Interestingly, lysosomes of the *C. elegans* mutants (Figure 2C, red arrows) showed quite similar ultrastructural features to the cultured hepatocarcinoma cells which were exposed to hydroxynonenal (Figure 2B, red arrows). 

**Figure 3:**
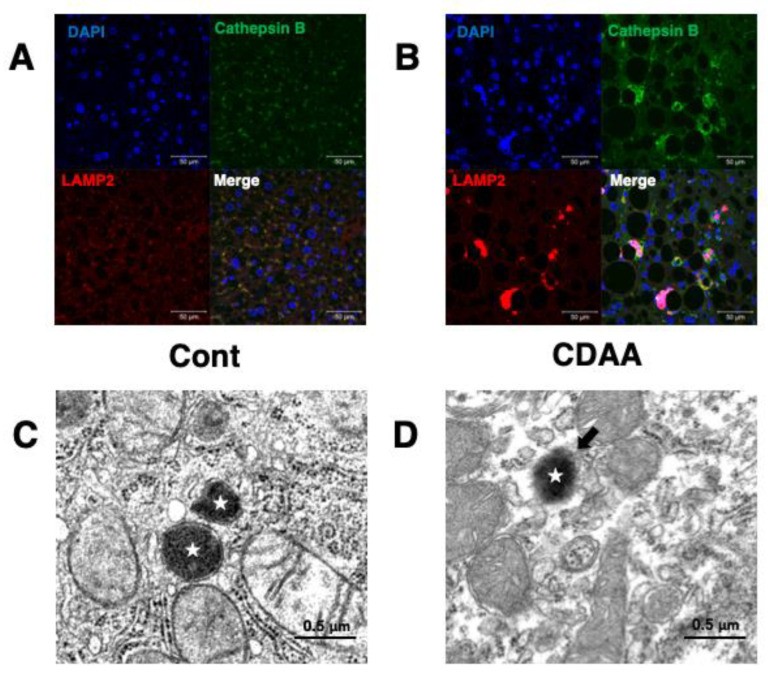
Lysosomal membrane permeabilization (LMP) in the liver tissue of NASH model mice. (**A**,**B**) Compared to the liver of the control mice being fed by the standard diet ((**A**) Cont), the liver of the NASH model mice being fed by the CDAA-diet ((**B**) CDAA), shows an enlarged area with the merged color of cathepsin B, LAMP2 and DAPI by immunofluorescence staining. Blue: DAPI, green; Cathepsin B, red: LAMP2. (**C**,**D**) Electron microscopic photographs of the liver tissue of the control mice (Cont) and CDAA mice (CDAA). The control mouse shows a distinct lysosomal membrane ((**C**) stars), whereas the CDAA mouse shows LMP ((**D**) arrow). Stars: lysosome. (Adapted from [18]).

**Figure 4:**
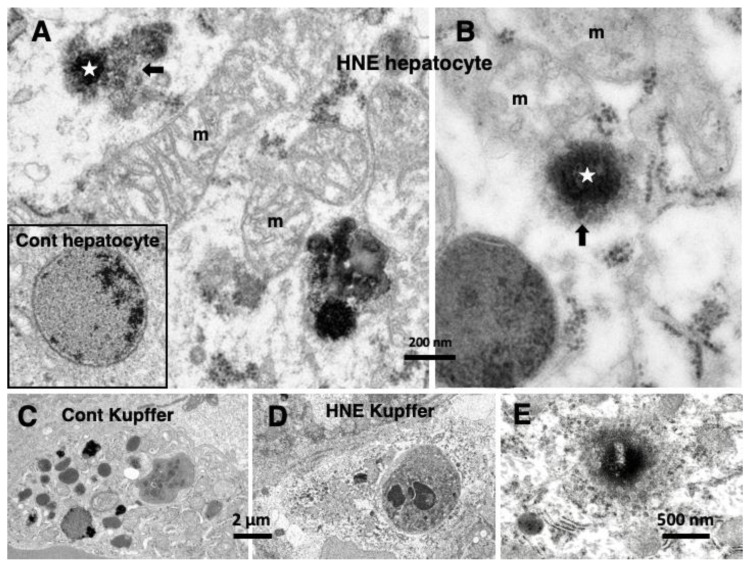
Electron microphotographs of the monkey liver after hydroxynonenal (HNE) injections (**A**–**D**) and the liver of human NASH patient (**E**). Compared to the membrane-bound lysosome of the control ((**A**) rectangle), the monkey hepatocytes after the hydroxynonenal injections show permeabilization ((**A**,**B**) arrows) of the lysosomal (stars)-limiting membrane. The degenerating lysosomes after hydroxynonenal injections often show shrinkage. Mitochondria show mild ((**A**) m) or severe ((**B**) m) disruption of the crista or double membrane, compared to the control. After the hydroxynonenal injections (**D**), lysosomal degradation is more severe in the Kupffer cells, compared to the sham-operated control (**C**). A liver biopsy specimen from a 60-year-old, female patient with NASH shows evidence of the lysosomal membrane permeabilization (**E**).

**Figure 5:**
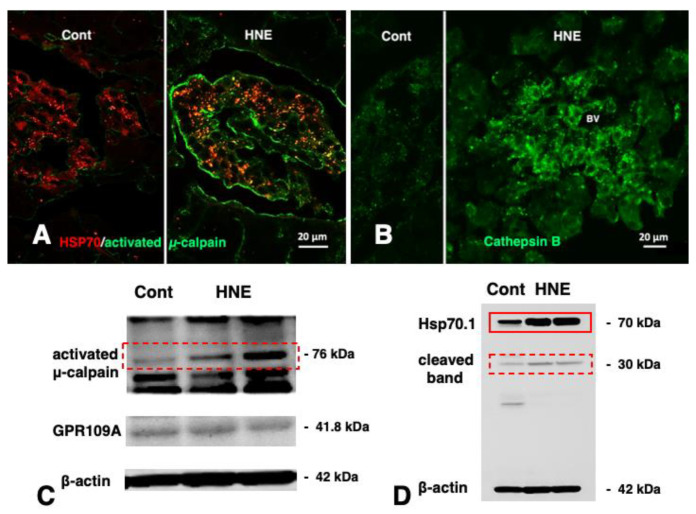
Calpain activation (**A**,**C**), Hsp70.1 cleavage (**D**), and cathepsin B leakage (**B**) in the Langerhans islets of the monkey pancreas after hydroxynonenal (HNE) injections. Activated μ-calpain immunoreactivity ((**A**) green) is negligible before hydroxynonenal injections ((**A**) Cont), whereas an increment of activated μ-calpain immunoreactivity occurs after hydroxynonenal injections ((**A**) HNE). After hydroxynonenal injections, activated μ-calpain immunoreactivity ((**A**) green) is colocalized with Hsp70.1 immunoreactivity ((**A**) red), showing a merged color ((**A**) HNE, yellow). This indicates interaction of activated μ-calpain with Hsp70.1, because the anti-μ-calpain antibody utilized here recognizes only the activated form but does not react with the inactivated form [14]. Cathepsin B immunoreactivity ((**B**) green) is stained as tiny granules in the control Langerhans islet ((**B**) Cont), whereas it is stained as coarse granules when associated with the cytoplasmic immunoreactivity after hydroxynonenal injections ((**B**) HNE), which indicates lysosomal membrane rupture/permeabilization. (BV; blood vessel) Western blotting shows that 76 kDa bands of activated μ-calpain increased after hydroxynonenal injections ((**C**) HNE) compared to the control ((**C**) Cont). Conceivably, hydroxynonenal contributed to activation of GPR109A (**C**) to induce Ca^2+^ mobilization necessary for μ-calpain activation. In response to the cell stress due to the hydroxynonenal injections ((**D**) HNE), not only Hsp70.1 main bands ((**D**) rectangle) but also cleaved Hsp70.1 bands of 30 kDa ((**D**) dot rectangle) are increased, compared to the control ((**D**) Cont). (Adapted from [30]).

**Figure 6:**
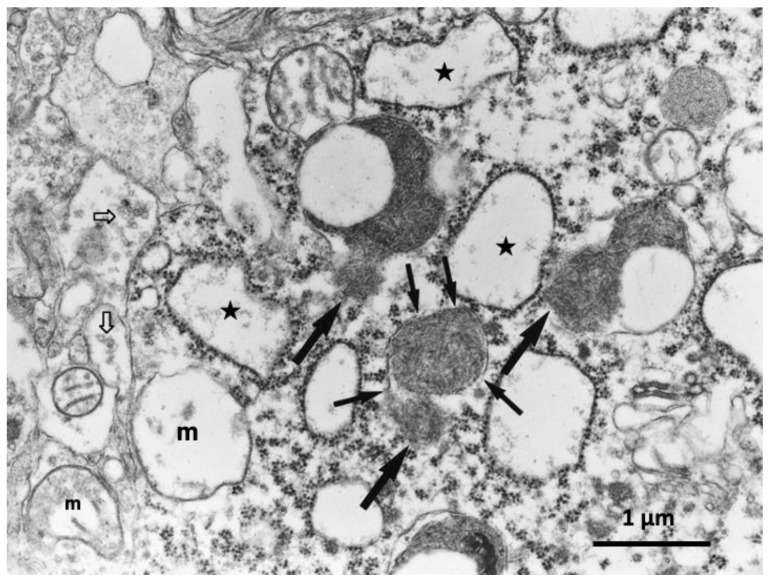
Electron microphotograph of the hippocampal CA1 neuron of a monkey after transient global brain ischemia. Lysosomes show rupture of the limiting membrane (thin arrows) with apparent leakage of the lysosomal content (thick arrows). Both swelling of rough ER (stars) and mitochondria (m) indicate intensity of the acute insult. The synaptic vesicles show a remarkable decrease (open arrows). This is the first electron microphotograph of the lysosomal membrane rupture reported ever (adapted from [14]).

**Figure 7:**
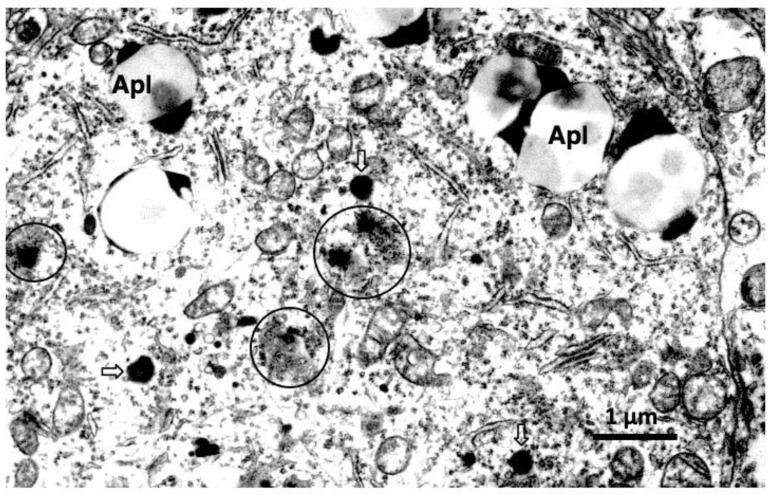
Electron microphotograph of the cortical neuron of a patient with Alzheimer’s disease. The Alzheimer neuron shows blurring of the limiting membrane of lysosomes with leakage of fine-granular contents and amorphous substance (circles). Ruptured lysosomes (circles) show a marked contrast with normal lysosomes (open arrows). Both increase of giant autophagolysosomes (Apl) and decrease of vivid lysosomes indicate long-standing degeneration in the Alzheimer neurons. (By the courtesy of Prof. Ralph A. Nixon, Nathan S. Kline Institute and New York University Langone Medical Center, New York).

Since the mammalian organs also showed similar lysosomal disintegrity as observed in the cultured cells Figure 1B and Figure 2B) and *C. elegans* mutants (Figure 2C), next we introduced experimental data of NASH model mice (Figure 3), monkeys after hydroxynonenal injections (Figure 4A–D and Figure 5) or after ischemic insult (Figure 6), as well as electron microphotographs of the human Alzheimer’s disease-affected brain (Figure 7) and NASH liver (Figure 4E).

## 4. CDAA-Diet-Fed NASH Model Mice 

To make the NASH model mice, male wild-type C57BL/6 mice were fed with choline-deficient, amino-acid defined (CDAA) diet for 8 weeks, according to the protocol of Koteish and Diehl [39]. Immunofluorescence histochemical staining of livers of the control mice being fed the standard diet (Cont), showed colocalization (merged color of yellow) of cathepsin B (green) and lysosome-associated membrane protein 2 (LAMP2) (red) as tiny granules around the DAPI-positive nucleus (Figure 3A, Merge). This means localization of cathepsin B within LAMP2-positive lysosomes. In contrast, in the NASH mice model being fed by the CDAA-diet (CDAA), both cathepsin B (green) and LAMP2 (red) immunofluorescence reactivities were enlarged, showing merged color of yellow or pink (Figure 3B, Merge). A triple color of pink was seen around the nucleus stained by DAPI. This indicated leakage of intra-lysosomal enzyme, cathepsin B, and scattering of lysosomal membrane protein, LAMP2, into the cytoplasm around lysosomes by the lysosomal membrane permeabilization/rupture. This was reinforced by the electron microscopic observation; lysosomes of the control (Cont) were bound by the limiting membrane (Figure 3C, stars), whereas LMP was suspected because of the blurred configuration of the lysosomal-limiting membrane in the hepatocytes of the NASH model mice (Figure 3D, star, arrow) [18].

## 5. Monkey Organ Damage after Hydroxynonenal Injections 

### 5.1. Liver

The serum concentration of hydoxynonenal in patients with Alzheimer’s disease is 20.65 μM/l at the median level (range: 6.02–25.20) [40]. The consecutive (5 mg/week × 12 weeks) injections of synthetic hydroxynonenal was carried out in Japanese macaque monkeys for reproducing this concentration. At autopsy 6 months after hydroxynonenal injections, the gross inspection of the liver showed a mosaic pattern of discoloring at the surface. The monkey liver was histologically characterized by widespread hepatocyte necrosis and lipid depositions [18,23,31]. The necrotic hepatocytes were microscopically characterized by an almost complete loss or marked disruption of organelles and nuclear chromatin. These histological changes were consistent with the characteristics of coagulation necrosis. 

Through electron microscopic observations, rough ER membranes were severely disrupted in the degenerating hepatocytes, and the mitochondria showed a marked dissolution of crista and double membrane (Figure 4A,B, m). Autophagosomes containing cell debris or damaged mitochondria were increased, whereas vivid lysosomes were remarkably decreased. After hydroxynonenal injections, the lysosomes of hepatocytes showed blurring of the limiting membrane, i.e., LMP (Figure 4A,B, stars), with leakage of its contents (Figure 4A,B, arrows). These showed a contrast to the intact lysosomes of the liver of the control monkeys, which were surrounded by the distinct limiting membrane (Figure 4A, rectangle). Compared to the change of lysosomes in the hepatocytes, the disintegration of lysosomes in the Kupffer cells was much stronger. Most of the lysosomes in the Kupffer cells faded away due to the rupture (Figure 4D), and this showed a remarkable contrast with the control which contained many lysosomes (Figure 4C). In response to hydroxynonenal, the lysosomal content of Kupffer cells was released into the sinusoid lumen. 

### 5.2. Pancreas

Through light microscopic observation, Langerhans islet cells in the same monkeys after hydroxynonenal injections showed scattered cell degeneration such as microcystic changes of the cytoplasm and dissolution of the nuclear chromatin [23,30]. Through electron microscopic observation, the degenerating β cells showed not only permeabilization of the lysosomal membrane but also rupture of the peroxisomal membrane. β cells showed a remarkable decrease of insulin granules and vivid lysosomes, which was associated with an increase of autophagosomes [30]. These were essentially similar to the changes in the liver after hydroxynonenal injections. 

Immunofluorescence histochemical staining showed a remarkable increase of Hsp70.1 immunoreactivity (red) which was colocalized with activated μ-calpain (green) in Langerhans islets after hydroxynonenal injections (Figure 5A, HNE, merged color of yellow). In the control, the cathepsin B immunoreactivity (green) was seen as tiny granules which were compatible with the size of intact lysosomes (Figure 5B, Cont). In contrast, after hydroxynonenal injections, cathepsin B immunoreactivity was seen as coarse granules with a slight degree of immunoreactivity throughout the cytoplasm (Figure 5B, HNE) [30]. This was considered evidence of lysosomal membrane disintegrity, being consistent with the immunohistochemical data of the brain (Figure 6 and Figure 7) and liver (Figure 3 and Figure 4). 

Western blotting showed an increase of 76 kDa band intensities of activated μ-calpain after hydroxynonenal injections (Figure 5C, dot rectangle). It is likely that activation of GPR109A (Figure 5C) by hydroxynonenal-induced Ca^2+^ mobilization might be sufficient for activating μ-calpain. In response to the stress of hydroxynonenal, the main bands of Hsp70.1 increased remarkably (Figure 5D, rectangle), while calpain-mediated Hsp70.1 cleavage [30], as shown by the 30 kDa bands (Figure 5D, dot rectangle), increased after hydroxynonenal injections, compared to the control. 

### 5.3. Brain 

The hippocampus is well known to be vulnerable to oxidative stress. Hippocampal CA1 neurons after consecutive hydroxynonenal injections, microscopically showed scattered neuronal death. Electron microscopic analysis of the degenerating CA1 neurons showed blurring of the lysosomal membrane with leakage of the intra-lysosomal content [31,41]. Interestingly, these ultrastructural changes induced by hydroxynonenal were similar to those caused by transient brain ischemia in monkeys (Figure 6) and the changes seen in the human brain with Alzheimer’s disease (Figure 7) or the human liver with NASH (Figure 4E), as shown below.

## 6. Monkey Brain after Transient Ischemia

After transient brain ischemia, the cathepsin immunoreactivity of the ischemia-resistant hippocampal CA 2–4 neurons was localized within granules of lysosomes in the non-stained, clear cytoplasm. In contrast, the cathepsin B immunoreactivity of the ischemia-vulnerable CA1 neurons was seen not only within the enlarged lysosomes but also throughout the cytoplasm. This finding indicated leakage of cathepsin enzymes from the lysosome into the cytoplasm in response to the insult of ischemia/reperfusion [14,21,25]. This was very similar to findings in the pancreas (Figure 5B) after hydroxynonenal injections. Consistent with the immunohistochemical findings, electron microscopic analysis of the CA1 neuron on days 3,4 after the ischemic insult showed rupture of the lysosomal-limiting membrane (Figure 6, thin arrows) which was associated with leakage of the lysosomal contents (Figure 6, thick arrows). This was ultrastructurally very similar to the lysosomal membrane rupture of *C. elegans* mutation models (Figure 2C, red arrows).

As the transient brain ischemia caused complete neuronal death on day 5 after the insult [14], at least a couple of days were necessary for the released hydrolytic enzymes to kill all CA1 neurons. Because of this time lag, it was not so difficult in the postischemic monkeys to find lysosomal membrane disintegrity by electron microscopy at the time points of days 3,4 after the insult. However, in the case of Alzheimer’s disease, it was extremely difficult to detect the lysosomal membrane disintegrity in the autopsy brain, because a large number of lysosomes had already faded away during the disease progression over decades. Conceivably, it would be easier to find evidence of lysosomal membrane rupture/permeabilization in the brain of patients with mild cognitive impairments. 

## 7. Human Alzheimer Brain and NASH Liver 

Among the electron microphotographs of the cortical neurons of the patients with Alzheimer’s disease, evidence of LMP with leakage of the lysosomal content (Figure 7, circles) was found [42], although it was extremely rare. The lysosomal membrane rupture/permeabilization was also confirmed in the hepatocyte of the human patients with NASH (Figure 4E, star) [18]. Alzheimer brain and NASH liver of human patients showed similar features to the liver of CDAA mice (Figure 3D) or monkey livers after hydroxynonenal injections (Figure 4). Importantly, evidence of the lysosomal membrane rupture/permeabilization was obtained beyond species, i.e., from *C. elegans* (Figure 2C) to mice (Figure 3), monkeys (Figure 4, Figure 5 and Figure 6), and humans (Figure 4E and Figure 7). 

## 8. Role of Lysosomal Membrane Proteins for Its Integrity

By forming a continuous carbohydrate layer at the luminal leaflet (glycocalyx), extensive glycosylation in the luminal portion of the lysosomal-limiting membrane proteins plays a protective role in the membrane [43,44]. The lysosomal membrane contains heavily glycosylated membrane proteins such as LIMP-1/CD63 and LIMP-2. Both may form a continuous carbohydrate layer at the luminal leaflet to prevent the limiting membrane from degradation by lysosomal acidic hydrolases [43]. LIMP-2 deficiency has been implicated in the pathogenesis of not only Gaucher disease but also Parkinson’s disease [45]. Since the *scav-*3 gene mutation in *C. elegans* (Figure 2C) induced similar lysosomal membrane rupture/permeabilization as seen in cell lines (Figure 1 and Figure 2), CDAA mice liver (Figure 3), monkey organs (Figure 4, Figure 5 and Figure 6), and human brain (Figure 7), the disorder of lysosomal membrane proteins may be crucial for the occurrence of the lysosomal membrane disintegrity beyond species. 

The potential role of μ-calpain for brain aging and Alzheimer’s disease was first reported by Nixon [46]. Subsequently, Taniguchi et al. found by Western blotting that μ-calpain is activated more than 7-fold in the brain tissues of Alzheimer’s disease brains, compared to age-matched, normal brains [47]. Calpains can be activated not only in vitro by amyloid β42 in the cultured rat neurons [48] but also in vivo by the insult of ischemia/reperfusion in the primate brain [14]. Like the acute, extensive ischemia during stroke, the long-term, mild ischemia due to arteriosclerosis associated with aging may also induce a certain degree of μ-calpain activation in patients in the prodromal stage of Alzheimer’s disease. This can lead to the cleavage of lysosomal membrane proteins such as Hsp70.1 [49], LAMP-2 [45,50,51], LIMP-2 (also known as SCARB2) [36,37,38], and/or subunit b2 of v-ATPase [52]. Since none of these proteins are related to the integrity of the lysosomal-limiting membrane in the physiological conditions, it is likely that cleavage of these proteins in the pathological conditions independently or synergically contributes to the occurrence of lysosomal membrane rupture/permeabilization [23]. 

## 9. Role of Hsp70.1 in the Calpain–Cathepsin Cascade 

Calpain activation at the lysosomal membranes may bring about programmed, necrotic cell death through the release of the hydrolytic cathepsin enzymes by the rupture of the limiting membrane. This cascade, formulated by the author’s group as the ‘*calpain-cathepsin hypothesis*’ in 1998 [25], consists of calpain-mediated disruption of the lysosomal membranes followed by the leakage of cathepsin enzymes into the cytoplasm. However, the target of activated μ-calpain had remained unelucidated. Ten years later, using proteomics analysis comparing normal and ischemic brain tissues of monkeys, Yamashima and his colleagues found that the target molecule of activated μ-calpain is a cell stress protein, Hsp70.1 (also called Hsp70 or Hsp72). Since Hsp70.1 has dual functions as a ‘chaperone protein’ and ‘lysosomal stabilizer’, they suggested that the calpain-mediated cleavage of the oxidized Hsp70.1 should be the cause of the lysosomal membrane disintegrity [23,49,53,54]. Accordingly, they focused on the specific oxidation, carbonylation of Hsp70.1, which is caused by hydroxynonenal. 

At home and in restaurants, deep-frying is a popular culinary method used worldwide, and the high oil content is a major health concern in many countries. For example, one of the most favorite fried foods in USA is French fries, and nearly half of potatoes produced are nowadays manufactured into French fries. Such high-oil and high-calorie affordable foods combined with a sedentary lifestyle can contribute to obesity, which is a major cause of prevalent chronic diseases, such as hypertension, type 2 diabetes, and NASH [29]. Especially, during deep-frying of vegetable oils made from rapeseed, soybean, sunflower, etc. that contain abundant amounts of linoleic acid, hydroxynonenal is generated and incorporated into the serum [23,55]. Further, even after incorporation into the body, reactive oxygen species attack ω-6 PUFAs in the membrane phospholipids to generate endogenous hydroxynonenal. As hydroxynonenal forms adducts with four different amino acids in proteins, namely cysteine (Cys), histidine (His), lysine (Lys), and arginine (Arg) residues, numerous proteins risk being modified by hydroxynonenal. 

**Scheme 1:**
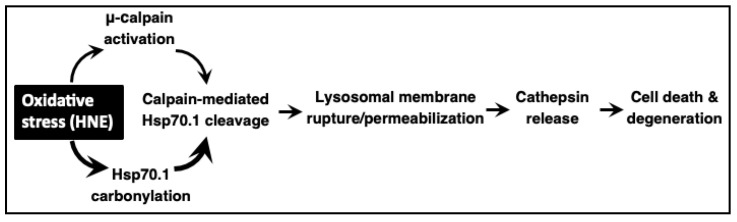
The ‘calpain-cathepsin hypothesis’ explaining the molecular cascade from vegetable oil-derived hydroxynonenal (HNE) to cell degeneration/death in diverse lifestyle-related diseases such as Alzheimer’s disease, type 2 diabetes, and nonalcoholic steatohepatitis (NASH).

For instance, in response to the ischemic insult, hydroxynonenal-induced ‘carbonylation’ occurs at the key site Arg469 of Hsp70.1 in the monkey brain [53]. A decrease of its molecular weight from 157.20 to 113.12 indicated implication of the specific oxidative injury of Hsp70.1. The same cascade of ‘calpain-mediated cleavage of the carbonylated Hsp70.1′ conceivably works not only in the monkey brain, liver, and pancreas but also in the human organs [18,23,30]. Hsp70.1, as a molecular chaperone and lysosomal stabilizer, is a stress-induced protein that confers cell protection against diverse stimuli. Calpain-mediated Hsp70.1 cleavage was demonstrated to occur in vitro following hydroxynonenal- or H_2_O_2_- mediated carbonylation, using diverse portions of the monkey brain tissues [56,57]. Since Hsp70.1 cleavage was blocked by the specific calpain inhibitor N-acetyl-Leu-Leu-Nle-CHO (ALLN) dose-dependently, it is likely that Hsp70.1 is a substrate of activated μ-calpain [22,49,57]. However, activated μ-calpain alone can insufficiently cleave the non-oxidized Hsp70.1, because hydroxynonenal-mediated carbonylation of Hsp70.1 plays a supportive but crucial role for facilitating the calpain-mediated Hsp70.1 cleavage (Scheme 1) [49]. The resultant Hsp70.1 dysfunction induces cell degeneration via the lysosomal membrane rupture/permeabilization. Using diverse experimental paradigms, the author and colleagues demonstrated that the same calpain-cathepsin cascade may work in the liver and pancreas in response to the oxidative injury [18,23,30,31]. If the timing of tissue sampling after insults or during disease is appropriate, this would be demonstrated in many experimental models and human diseases.

Overall, the lysosomal membrane contains more than 100 proteins, including lysosomal-anchoring proteins, transporters, receptors, and enzymes [58]. Recent studies have uncovered a range of lysosomal membrane proteins that influence lysosomal cell death [32], and this field of research is expanding and constantly yielding new insights for elucidating the etiology of diseases. Especially, the risk of using cooking oils should be more and more paid attention to as a source of a chronic oxidative stressor, hydroxynonenal. The influence of reactive oxygen species, calpain, and hydroxynonenal upon lysosomal membrane proteins should be studied further to elucidate the molecular mechanism of lysosomal membrane rupture/permeabilization. The author speculates that the calpain-mediated cleavage of the lysosomal membrane proteins including Hsp70.1 may be crucial for the lysosomal cell death, although this is very difficult to confirm in alive patients with lifestyle-related diseases. 

## 10. Conclusions

In this review, the author provided evidence of electron microphotographs showing lysosomal membrane disintegrity occurring in several species, from *C. elegans* to primates, in diverse pathologic states and diseases. Lysosomal membrane rupture/permeabilization may occur within minutes or hours, or at the longest, within days, and we have, if any, a very restricted chance of encountering a very small number of residual lysosomes in the dying cells at the given time point. Accordingly, careful and detailed electron microscopic observations are indispensable to find the exact cause of diseases. Most importantly, the peroxidation product of linoleic-acid-rich vegetable oils, hydroxynonenal, induces Hsp70.1 disorders with the resultant lysosomal membrane disintegrity, facilitates cell degeneration/death, and contributes to the occurrence of lifestyle-related diseases. We should keep in mind that ultrastructural evidence of the lysosomal membrane disintegrity can be found not after cell death or disease is completed, but found more easily during the progression phase of cell degeneration or the prodromal stage of a disease.

## Data Availability

Permissions were obtained from Rockfeller University press (Figure 2C), Elsevier (Figure 3, Supplementary Movie), and Wiley (Figure 6).

## Supplementary Materials

The following supporting information can be downloaded at https://www.mdpi.com/article/10.3390/nu15030609/s1.

## Abbreviations

ALLN	N-acetyl-Leu-Leu-Nle-CHO
*C. elegans*	*Caenorhabditis elegans*
CDAA-diet	choline-deficient, amino-acid defined-diet
CA1	*cornu Ammonis* 1
Hsp70.1	heat-shock protein 70.1
DAPI	4’,6-diamidino-2-phenylindole
HNE	hydroxynonenal
LAMP-2	lysosome-associated membrane protein 2
LIMP-2	lysosomal integral membrane protein type 2
LMP	lysosomal membrane permeabilization
NASH	nonalcoholic steatohepatitis
PUFA	polyunsaturated fatty acid
zVAD-fmk	Z-Val-Ala-Asp-fmk
