# Supplementary material for: Implication of Vegetable Oil-Derived Hydroxynonenal in the Lysosomal Cell Death for Lifestyle-Related Diseases
Source: Nutrients. 2023 Jan 24;15(3):609. doi: 10.3390/nu15030609 (PMC9921130; doi:10.3390/nu15030609)
Supplement: Supplementary file 1 [file nutrients-15-00609-s001.zip › nutrients-2146041-supplementary.pdf]

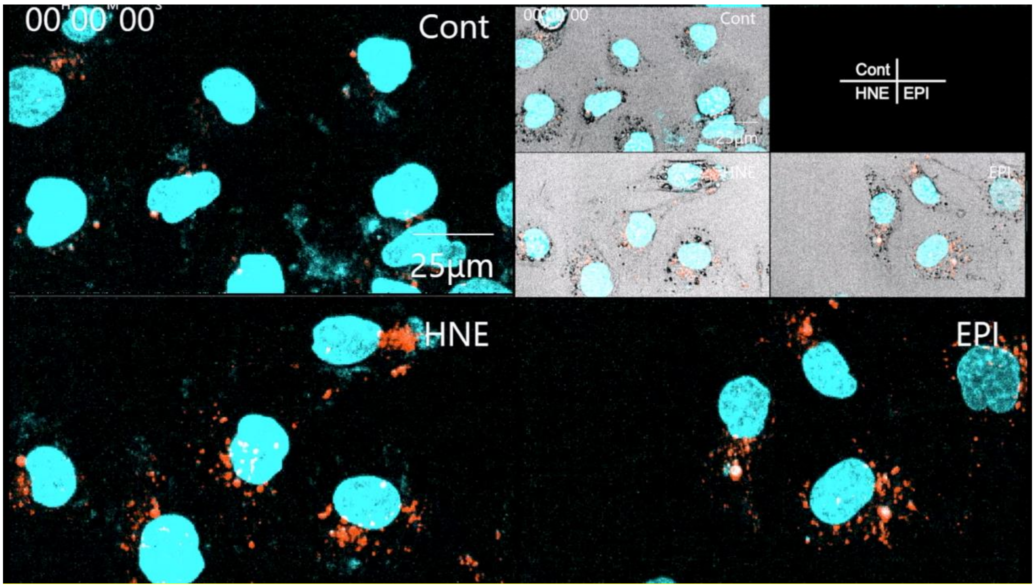

Supplementary Video S1: lysosomal rupture in HNE-induced necrosis, but not EPI-induced apoptosis. A fluorescence timelapse imaging movie of HepG2 hepatoma cell lines with the addition of either hydroxynonenal (HNE, lower left column) or anti-cancer agent, epirubicin hydrochloride (EPI, lower right column). The upper right column shows dark field imaging to show the outcome of lysosomes being stained orange by LysoTracker. The lower column shows light field imaging to show the cell death pattern. The alive nucleus is intensely stained blue by Hoechst 33,342, while the dead nucleus shows negligible staining with Hoechst but is intensely stained orange by propidium iodide in the EPI-induced apoptosis. In the HNE-induced necrotic cell death (left), lysosomal rupture (orange) is observed in the early phase of cell degeneration, whereas in the EPI-induced apoptotic cell death (right), lysosomes remain grossly intact until the late phase. Black granules, presumably indicating mitochondria, show distinct outcome between HNE- and EPI-induced cell death. Mitochondria disappear in the former in the early phase of cell degeneration (left), while they remain grossly intact for a long time in the latter (right). (Cited from Seike et al., Cell Mol Gastroenterol Hepatol. 14:925–944, 2022. https://doi.org/10.1016/j.jcmgh.2022.06.008). https://www.cmghjournal.org/cms/10.1016/j.jcmgh.2022.06.008/attachment/7a31d47d-8c15-4895-99dd-e9952ce5a887/mmc3.mp4.
